# Subtype Distribution of *Blastocystis* Isolates in Sebha, Libya

**DOI:** 10.1371/journal.pone.0084372

**Published:** 2013-12-20

**Authors:** Awatif M. Abdulsalam, Init Ithoi, Hesham M. Al-Mekhlafi, Abdulsalam M. Al-Mekhlafi, Abdulhamid Ahmed, Johari Surin

**Affiliations:** 1 Department of Parasitology, Faculty of Medicine, University of Malaya, Kuala Lumpur, Malaysia; 2 Department of Medical Laboratory Sciences, Faculty of Engineering and Technology, University of Sebha, Sebha, Libya; 3 Department of Parasitology, Faculty of Medicine and Health Sciences, Sana’a University, Sana’a, Yemen; 4 Department of Biology, Faculty of Natural and Applied Sciences, Umaru Musa Yar’adua University, Katsina, Nigeria; Kliniken der Stadt Köln gGmbH, Germany

## Abstract

**Background:**

*Blastocystis* is a genetically diverse and a common intestinal parasite of humans with a controversial pathogenic potential. This study was carried out to identify the *Blastocystis* subtypes and their association with demographic and socioeconomic factors among outpatients living in Sebha city, Libya.

**Methods/Findings:**

*Blastocystis* in stool samples were cultured followed by isolation, PCR amplification of a partial SSU rDNA gene, cloning, and sequencing. The DNA sequences of isolated clones showed 98.3% to 100% identity with the reference *Blastocystis* isolates from the Genbank. Multiple sequence alignment showed polymorphism from one to seven base substitution and/or insertion/deletion in several groups of non-identical nucleotides clones. Phylogenetic analysis revealed three assemblage subtypes (ST) with ST1 as the most prevalent (51.1%) followed by ST2 (24.4%), ST3 (17.8%) and mixed infections of two concurrent subtypes (6.7%).

**Blastocystis:**

ST1 infection was significantly associated with female (*P* = 0.009) and low educational level (*P* = 0.034). ST2 was also significantly associated with low educational level (*P*= 0.008) and ST3 with diarrhoea (*P* = 0.008).

**Conclusion:**

Phylogenetic analysis of Libyan *Blastocystis* isolates identified three different subtypes; with ST1 being the predominant subtype and its infection was significantly associated with female gender and low educational level. More extensive studies are needed in order to relate each *Blastocystis* subtype with clinical symptoms and potential transmission sources in this community.

## Introduction

*Blastocystis hominis* is an enteric unicellular parasite of humans and many animals. It is classified taxonomically within the heterogeneous group of the Stramenopiles [1]. *Blastocystis* infections have a worldwide distribution with prevalence of 30% to 60% in developing countries and 1.5% to 20% in developed countries [2,3]. These differences are due to poor hygiene practices and consumption of contaminated food or water [2,4,5]. The organism is mainly transmitted through the faecal-oral route [6]. A higher risk of infection has been found in humans with close animal contact and several studies provided molecular-based evidence on the zoonotic potential of *Blastocystis* sp. [7,8,9,10]. *Blastocystis* is currently classified into 17 small subunit ribosomal RNA (SSU rDNA) subtypes (STs; ST1–17) according to the nomenclature established by Stensvold et al. [11]. These subtypes represent genetically diverse *Blastocystis* species isolated from humans and animals. A majority of human infections with *Blastocystis* sp. are attributable to ST3, but infections with ST1, ST2 and ST4 are also common [12,13]. ST5-ST9 have been isolated only sporadically from humans [9,14,15] while ST10–ST17 have not been found in humans to date [8,16,17,18,19]. 

The pathogenic potential of *Blastocystis* is controversial because the infection can be asymptomatic. However, accumulating epidemiological data strongly suggest that *Blastocystis* is an emerging pathogen [20]. The controversial pathogenesis of *Blastocystis* may attribute to subtype variations in virulence thus explaining the variability in symptoms observed in *Blastocystis* patients. Several recent reports suggested that ST1, ST4, and ST7 were pathogenic [21,22,23] whereas ST2 and ST3 consist of both pathogenic and non-pathogenic parasites [24,25]. Infection with *Blastocystis* is believed to be associated with gastrointestinal symptoms including acute or chronic diarrhoea, abdominal pain, flatulence, anorexia, nausea and vomiting [26,27,28]. Beside, this parasite may also play a significant role in several chronic gastrointestinal illnesses such as irritable bowel syndrome and inflammatory bowel disease [29,30,31]. In Libya, studies on *Blastocystis* are limited and most of available data pertaining to human *Blastocystis* were derived from direct faecal smear examination [32,33,34,35]. Therefore, this study represents the first investigation of *Blastocystis* subtypes and its associated factors in Libyan outpatients.

## Materials and Methods

### Ethics statement and patient record

The protocol of this study was approved by the Medical Ethics Committee of University Malaya Medical Centre (MEC Ref. No: 782.11), Kuala Lumpur, Malaysia. Permission to conduct this study was also given by the Faculty of Engineering and Technology, University of Sebha and Sebha Central laboratory authorities (Ref. No: 533/2010). Prior to data collection, the objectives, the possible advantages and disadvantages of this study were explained to the participants. After a clear explanation, a written consent was then obtained from each of volunteer, as well as from parents or guardians on behalf of their children. Each volunteer was then given a 100 mL volume wide-mounted and spoon-screw-capped container, in which to put a faecal specimen and bring to the Sebha Central laboratory. Only volunteers who gave their consents and were able to provide fresh stool specimens were included in the study. Upon receiving the stool specimens, patient’s personal information and complaints were collected using a standardized questionnaire. On the basis of patient records, *Blastocystis*-positive individuals were characterized as ‘asymptomatic’ without any gastrointestinal (GI) symptoms or ‘symptomatic’ with GI symptoms including abdominal pain, diarrhoea (≥3 loose or watery stools per day), flatulence, constipation, nausea and vomiting. Data collection and analysis of faecal specimens were carried out between August and November 2010.

### Collection of stool samples and cultivation of *Blastocystis*

This cross-sectional study was carried out in Sebha city, about 800 km south of Tripoli (longitude 14.42°E, latitude 27.03°N), Libya. The area is characterized by desert climate, dry and hot weather and low rainfall. Agriculture is the main occupation of the people and underground wells are the main source of water for drinking and household use. Detailed description of the study area and population has been published previously [36]. Stool samples were obtained from symptomatic and asymptomatic individuals of outpatients visiting the Sebha Central Laboratory. Screening for *Blastocystis* and other intestinal parasites was performed immediately upon receiving the stool specimens by using direct smear preparation, xenic in vitro culture (XIVC), formalin ethyl acetate concentration, trichrome stain and modified Ziehl-Neelsen stain. Investigation on the potential viral or bacterial infection was not carried out in this study. 

The xenic in vitro culture (XIVC) is used as a standard method for diagnosis of Blastocytis in Para :SEAD Laboratory (a diagnostic division of the Department of Parasitology, University Malaya Medical Centre, Malaysia) for more than 15 years. Recently, XIVC has been reported to be more sensitive in detecting *Blastocystis* although it is not commonly used in the diagnostic laboratory [37,38,39]. For each culture, approximately 50 mg of stool was inoculated into a 15-mL screw-cap tube containing 5 mL of Jones’ medium supplemented with 10% horse serum. Four replicates of inoculation were carried out for each stool sample. All inoculated tubes were tightly-closed, placed in a rack and incubated at 37°C for 2-3 days. The presence of *Blastocystis* sp. was observed daily for 14 days of cultivation, by placing one drop of the cultured sediment onto a glass slide, covered with a cover slip and viewed (X100 and X400 objectives) under light microscopy. Positive cultures were defined by the detection of any form of *Blastocystis* sp. (i.e. vacuolar, granular and amoeboid forms). Positive cultures were subsequently sub-cultured into fresh complete Jones medium once every two days. Each of *Blastocystis* positive XIVC was packaged approximately 1-2 x 10^4^ cells in 10 ml of complete Jones’ medium, in a tightly closed culture tubes and shipped to Malaysia by air. 

### DNA extraction

*Blastocystis* cells were sub-cultured in the laboratory of the Department of Parasitology, University Malaya Medical Centre, Malaysia. Approximate 1×10^6^ *Blastocystis* cells were harvested from cultures by centrifugation at 500×g for 5 min. The pellet was washed with phosphate-buffered saline (pH 7.4) for five times using centrifugation. The clean cells were subjected to extraction of genomic DNA with the DNAzol® Reagent (Invitrogen, USA) following the manufacturer’s instructions.

### PCR amplification

For each sample, 2-5 μL (20 ng/μL) genomic DNA was subjected to PCR analysis using the forward Blast 505–532 (5’ GGA GGT AGT GAC AAT AAATC 3’) [40] and reverse Blast 998–1017 (5’TGC TTT CGC ACT TGT TCATC 3’) [41] primers, targeting the small subunit (SSU) rDNA gene of *Blastocystis*. PCR amplification was performed in a 50-μL volume per reaction using 1 μM of each primer (Bioneer, South Korea), 1× PCR buffer, 1.5 mM MgCl_2_, 0.2 mM dNTP, 2.5 U Taq (Fermentas) and 2.5 μL BSA (0.1 g/10 mL) (New England Biolabs, USA). The PCR amplification consisted of 35 cycles of 95°C for 30 sec, 54°C for 30 sec and 72°C for 30 sec after an initial pre-heat step at 95°C for 4 min. A final extension step at 72°C for 5 min was included [41]. The PCR products were separated by agarose gel electrophoresis, and bands of the expected size (approximately 500 bp) were purified using the QIAgen Gel Extraction Kit (QIAGEN, USA) according to the manufacturer’s protocol.

### Cloning and sequencing

Purified PCR products were cloned in the pGEM®-T Vector (Promega, USA) and amplified in *Escherichia coli* JM109 competent cells. Recombinant clones were selected from each specimen and screened by PCR. Minipreparations of plasmid DNA were done using the QIAprep Spin Miniprep kit (QIAGEN, USA). Three or four clones containing inserts of approximately the expected size were arbitrarily selected for each sample and sequenced on both strands using M13 forward (5’GTA AAA CGA CGG CCA GT 3’) and reverse (5’GCG GAT AAC AAT TTC ACA CAG G 3’) primers. Cycle sequencing was carried out using the ABI BigDye^®^ Terminator Cycle Sequencing Ready Reaction Kit v3.1, using the ABI PRISM^®^ 3730xl DNA Analyzer (Applied Biosystems, USA). Sequences obtained in this study have been deposited in Genbank under accession numbers KF306282 to KF306295.

### Phylogenetic analysis

Chromatogram sequences belonging to cloned DNA fragment were extracted from the vector using Gene Runner software (version 3.05). These sequences were then subjected to BLAST searches in the Genbank database to confirm isolates as *Blastocystis* sp. The subtypes were identified by determining the exact match or closest similarity against all known *Blastocystis* subtypes according to the last classification by Stensvold et al. [11]. The published sequences from Genbank and those obtained from this study were aligned using Clustal W property implemented in BioEdit software (version 7.0.9.0) [42]. The phylogenetic tree was then constructed with neighbor-joining (NJ) analysis of SSU rDNA using the MEGA version 4 software [43], and molecular distances were estimated by Kimura two-parameter model [44]. Branch reliability was assessed using bootstrap analysis (1000 replicates). *Proteromonas lacerate* (U37108) was used as the out-group.

### Statistical analysis

Statistical analysis was performed using the *Statistical Package for Social Sciences for Windows* (SPSS), version11.5 (SPSS Inc, Chicago, IL, USA). Pearson’s Chi-square (χ^2^) or Fisher’s exact test was used where appropriate to test the associations between *Blastocystis* subtypes and associated factors. All *P* values < 0.05 were considered statistically significant.

## Results

### Description of test isolates

A total of 380 stool samples were collected from the Libyan outpatients, which consisted of 197 males and 183 females, aged from 1 to 75 years (median age = 25 years, inter-quartile range = 36 years). *Blastocystis* was detected in 84 (22.1%) samples by using microscopy after *in vitro* cultivation. On analysis, 54 and 30 of these samples were from symptomatic (abdominal pain, flatulence diarrhoea, constipation, nausea and vomiting) and asymptomatic patients, respectively. The general characteristics of the outpatients are shown in Table S1. 

Only 64 samples were subjected for PCR amplification as the remaining (20/84) died off during subsequent sub-cultures. Out of the 64 isolates, 86.0% (55/64) successfully produced the expected size of 500 bp fragment of the SSU rDNA after PCR amplification. Of these, 45 isolates were successfully cloned in *Escherichia coli* JM109 and readable DNA sequences were obtained at approximately 479-483 bp of the *Blastocystis* SSU rDNA gene. After the BLAST search, all of these isolate clones sequences showed a high homology (98.3-100%) with their closest matching reference sequences of *Blastocystis* from Genbank. Concurrently, these 45 isolates were from 45 patient’s stool samples, of which 43 were detected only *Blastocystis* and 2 (L5-27/M and L18-9/F) were co-infected with *Cryptosporidium* and *Giardia intestinalis*, respectively (Table S2).

### Phylogenetic analysis and multiple alignments

**Figure 1:**
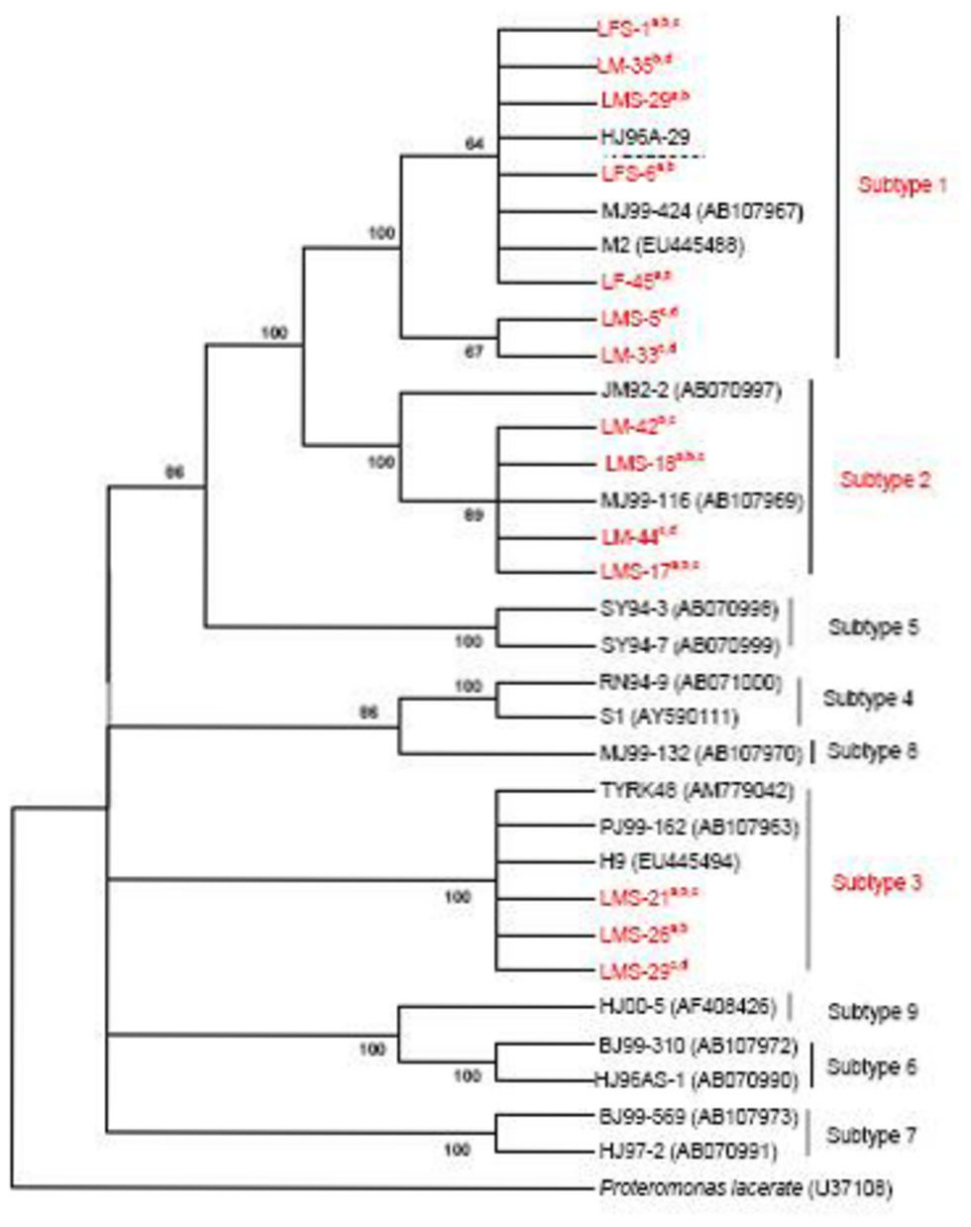
Phylogenetic tree of the SSU-rDNA gene sequences of *Blastocystis* isolates as inferred using the neighbour-joining method. Isolate or strain names are as provided in Genbank as available, followed by accession numbers in parentheses. Sequences generated in this study (designated ‘LM(S), LF(S)’ followed by clones ‘^a,b,c,d^’) are in red font. *Proteromonas* *lacerate* (accession number U37108) served as the out-group. Bootstrap values (%) are indicated at the internal nodes (1,000 replicates). Bootstrap values of less than 50% are not shown.

The isolate clones sequences were detected to be clustered under three respective subtypes after phylogenetic analysis with the selected reference isolates of *Blastocystis* assemblage subtypes ST1 to ST9 (Table 1). Phylogenetic tree was constructed using the stramenopile *Proteromonas lacerate* as the out-group (Figure 1). The rooted neighbor-joining tree identified nine clades that corresponded to ST to ST9, and each subtype was shown strongly supported by high bootstrap values. The clones that were grouped in the same subtype clustered with each other with good bootstrap support, and therefore the three respective subtypes (ST1, ST2, and ST3) were seen as three independent monophyletic groups. ST1, ST2 and ST3 formed a group of the triplicate clone (a,b,c) sequences with identical nucleotides representing 19, 10 and 7 isolates, respectively (Table S2). The other 9 isolates consisted of clones sequences with non-identical nucleotides belonging to either the same or different *Blastocystis* subtypes. In general, 42 isolates were of a single subtype, where 23, 11, and 8 were under ST1, ST2 and ST3, respectively. Another 3 isolates were detected to be a mix of two different subtypes; one (LMS-29) consists of ST1 and ST3, and the other two (LM-44, LF-45) consist of ST1 and ST2, respectively (Table S2). 

**Table 1:** Genbank reference sequence of *Blastocystis* isolates used in the construction of phylogenetic tree.

Subtype	Accession number	Host	Reference
ST1	AB070989	Human	[48]
	AB107967	Monkey	[70]
	EU445488	Monkey	[71]
ST2	AB070997	Monkey	[48]
	AB107969	Monkey	[70]
ST3	AM779042	Human	[12]
	AB107963	Pig	[70]
	EU445494	Human	[71]
ST4	AB071000	Rat	[48]
	AY590111	Rat	[7]
ST5	AB070998	Pig	[48]
	AB070999	Pig	[48]
ST6	AB107972	Bird	[70]
	AB070990	Human	[48]
ST7	AB070991	Human	[48]
	AB107973	Bird	[70]
ST8	AB107970	Primate	[70]
ST9	AF408426	Human	[55]

Multiple pair-wise alignments of DNA sequences among isolate clones were performed against the SSU rDNA gene of their closest match reference strains from Genbank represented by *Blastocystis* ST1, ST2 and ST3. The DNA sequences of isolate clones that showed 100% homology with their closest match reference were only seen in ST1 and ST3. However, up to seven bases polymorphism due to base insertion, substitution and deletion were seen in all isolate clones belonging to ST2; six groups of clones [(LMS-5^cd^) (LFS-6^a,b^, LM-35^a,c^, LMS-29^a,b^) (LM-33^c,d^) (LFS-6^c,d^, LM-35^b,d^) (LF-45^a,b^, LM-44^a,b^) (LMS-29^ab^)] belonging to ST1 and two groups [(LMS-26^ab^) (LMS-29^cd^)] belonging to ST3 (Table S2 and Table 2).

**Table 2:** Heterogeneity of *Blastocystis* clone against selected closest match reference from Genbank.

Isolate (clones) from symptomatic and asymptomatic	Sequence	BI	BS	BD	No. of	ST	Reference isolate
patients	homology				BI/BS/BD		(Accession no.)
LFS-1 (a,b,c) (other 20 isolate clones stated in Table S2)	100%					1	MJ99-424 (AB107967);
LMS-5 (c,d)	99.7%		G^280^ →C		1BS		J96A-29, (AB070989)
LM-33 (c,d)	99.7%		G^280^ →A		1BS		
LM-44 (a,b), LF-45 (a,b)	99.7%		C^36^→A		1BS		
LMS-29 (a,b)	98.9%	- ^194^→T		C^188→ -^	1BI, 1BD		
LMS-18 (a,b,c), LM-19 (a,b,c), LFS-20 (a,b,c),	98.9%	-^199^→C	T^208^→C	T^193→ -^	2BI, 1BS,2BD		
LM-40 (a,b,c), LF-41 (a,b,c), LM-42 (a,d)		- ^200^→C		T ^216→ -^			
LFS-6 (a,b), LM-35 (a,c)	98.7%	- ^193^→C		C^188^ → -	1BI, 1BD		
LM-42 (b,c)	98.7%	-^199^→C	T^208^→C	T^193→ -^	2BI, 2BS,2BD		
		- ^200^→C	C^230^→T	T ^216→ -^			
LFS-6 (c,d), LM-35 (b,d)	98.5%	- ^193^→C	T^209^→A	C^188→ -^	1BI, 1BS,1BD		
LMS-16 (a,b,c), LMS-17 (a,b,c), LM-39 (a,b,c)	99.7%		T^207^→C		1BS	2	MJ99-116(AB107969)
LM-43 (a,b,c)							
LM-44 (c,d), LF-45 (c,d)	98.3%	- ^198^→T	T^36^→C	T^193→ -^	2BI, 3BS,2BD		
		**^*-*^** ^ 200 ^ **^*→*^**G	T^208^→C	T ^216→ -^			
			A^467^→G				
LMS-21 (a,b,c) (other 7 isolate clones stated in Table S2)	100%					3	PJ99-162(AB107963)
LMS-29 (c,d)	99.7%		A^222^→T		1BS		
LMS-26 (a,b)	99.1%		G^214^→T		4BS		
			T^215^→G				
			C^220^→A				
			T^274^→A				

Designation: Clone (a,b,c,d), base insertion (BI), base substitution (BS), base deletion (BD), subtype of *Blastocystis* (ST), *Blastocystis* isolate from Libyan female (LF), female with symptom (LFS), male (LM), male with symptom (LMS), guanine (G), cytosine (C), adenine (A), thymine (T), base deletion/insertion position (-). Sequence of isolate clones (bold font) and the reference isolates (accession number) were used in the phylogenetic analysis. 

### Subtype and related factors

*Blastocystis* ST1 was the most prevalent among Libyan outpatients (51.1%, 23/45), followed by ST2 (24.4%, 11/45) and ST3 (17.8%, 8/45). In addition, 6.7% (3/45) were mixed infections consisting of two subtypes, which are either ST1 and ST2 or ST1 and ST3. Concurrently, ST1 and ST2 were detected in both asymptomatic and symptomatic patients, whereas all patients with ST3 presented with gastrointestinal symptoms (Table S2). The association between *Blastocystis* ST1, ST2 and ST3 infections and some demographic and socioeconomic factors was appropriately analysed either by Chi-square test or Fisher’s exact test and the results are presented in Table 3. *Blastocystis* ST1 infection was significantly associated with the female gender (*x* ^2^ = 6.736; *P* = 0.009) and low educational level (*x* ^2^ = 4.325; *P* = 0.034). Infection with *Blastocystis* ST2 was significantly associated with low educational level (Fisher’s exact test; *P* = 0.008). ST3 was significantly associated with diarrhoea (Fisher’s exact test; *P* = 0.008). No significant associations were found between infections with any of the observed *Blastocystis* subtypes and age group, working status, drinking water (treated or untreated) or the presence of animal in the household.

**Table 3:** Association between *Blastocystis* subtypes and related potential factors.

Variable	ST1		ST2		ST3	
	Frequency (%)	*P* ^a^	Frequency (%)	*P* ^b^	Frequency (%)	*P* ^b^
Gender		0.009 ^*^		0.080		0.431
Female	14 (60.9)		3 (27.3)		2 (25.0)	
Male	9 (39.1)		8 (72.7)		6 (75.0)	
Age		0.056		0.123		NA
≥18 years	18 (78.3)		9 (81.8)		8 (100.0)	
<18 years	5 (21.7)		2 (18.2)		0 (0.00)	
Education level		0.034^*^		0.008^*^		0.932
Low (≤ Primary school)	18 (78.3)		9 (81.8)		5 (62.5)	
High (≥Secondary school)	5 (21.7)		2 (18.2)		3 (37.5)	
Occupational status		0.801		0.987		0.764
Not working	13 (56.5)		6 (54.5)		4 (50.0)	
Working	10 (43.5)		5 (45.5)		4 (50.0)	
Family size		0.711		0.974		0.709
≥ 7 members (large)	12 (52.2)		6 (54.5)		5 (62.5)	
< 7 members (small)	11 (47.8)		5 (45.5)		3 (37.5)	
Presence of animals in the house		0.488		0.353		0.725
Yes	10 (43.5)		7 (63.6)		3 (37.5)	
No	13 (56.5)		4 (36.4)		5 (62.5)	
Drinking water		0.204		0.867		0.243
Untreated	13 (56.5)		5 (45.5)		4 (50.0)	
Treated (chlorinated, filtered or boiled)	10 (43.5)		6 (54.5)		4 (50.0)	
Diarrhoea		0.480		0.303		0.008^*^
Yes	8 (34.8)		4 (36.4)		7 (87.5)	
No	15 (65.2)		7 (63.6)		1 (12.5)	
Abdominal pain		0.384		0.384		0.700
Yes	10 (43.5)		6 (54.5)		4 (50.0)	
No	13 (56.5)		5 (45.5)		4 (50.0)	
Flatulence		0.270		0.270		0.686
Yes	7 (30.4)		5 (45.5)		3 (37.5)	
No	16 (69.6)		6 (54.5)		5 (62.5)	

^a^ Chi square test, ^b^Fisher’s Exact test, ^*^Significant association (*P*<0.05), not applicable (NA).

## Discussion

In the present study, the overall prevalence of *Blastocystis* was 22.1% (84/380). Of these, 64 isolates were subjected to PCR amplification and in 86.0% (55/64) the expected 500 bp fragment of the SSU rDNA was successfully amplified. In 9 instances, no PCR product was produced despite repeated DNA extractions and several attempts towards optimising PCR conditions. Possible reasons for this could be due to PCR inhibition. A previous molecular study found that PCR failed to detect 25% of samples that were positive by culture due to PCR inhibitors in stool samples [13]. It is also possible that this specimen contained an isolate not amplifiable by the primers [45]. Indeed, the choice of PCR primers may influence the ability to successfully detect the parasite with the possibility that some primers are more subtype-specific. Furthermore, some primers work well with cultured isolates, whereas others work well with DNA extracted directly from faeces. Because of the possibility that not all *Blastocystis* STs have been identified, it is advisable to use multiple primer pairs, or to develop multiplex PCR analyses for characterizing *Blastocystis* isolates [46]. Of 55 PCR-positive samples, 45 isolates were successfully cloned and readable DNA sequences were obtained. For the other 10 PCR positive isolates (10/55), the recombinant clones could not be obtained despite numerous attempts toward optimizing the cloning reaction. This could be due to the presence of an inhibitory component in the PCR product or damage to the DNA caused by UV light during excision from the agarose gel. Thus, preparing a new DNA template was necessary; however, this was not done for these samples as it was not possible to collect new samples from related patients. 

There was no nucleotide differences found among the 36 out of 45 *Blastocystis* isolates. In 9 instances, nucleotide differences were detected among the three groups of clones. Of these, 3 isolates (LMS-29, LM-44 and LF-45) from each L27-45/M, L3-20/M and L4-33/F patient were mixed infections containing two different *Blastocystis* subtypes. The difference between the two groups of duplicate clones in the similar isolate is likely due to the co-infection with two variants within the same subtype or sequence variations between SSU rDNA gene copies within the same isolate [47]. The sequence heterogeneity (intra- and inter-subtype) of the SSU rDNA genes among *Blastocystis* isolates were reported in many previous studies [7,48,49,50].

The DNA sequences of isolate clones assemblage *Blastocystis* ST1 revealed 98.5% to 100% identity with the reference *Blastocystis* isolate MJ99-424 (AB107967) and HJ96A-29 (AB070989). Intra-subtype sequence polymorphism was due to a single base substitution, an insertion, or a deletion. For ST2, all isolate clones showed nucleotide polymorphism (98.3 - 99.7% homology) in comparison with the reference isolate MJ99-116 (AB107969), with up to 3 base substitutions, 2 insertions and 2 deletions. Subsequently, isolate clones assemblage ST3 showed 99.1% to 100% identity with the reference isolate PJ99-162 (AB107963); sequence polymorphism was shown only in isolate clones LMS-29^cd^ and LMS-26^ab^ with one and four base substitutions, respectively. Moreover, intra-subtype sequence polymorphism may naturally reflect the potential existence of subgroups within the same subtype as recently suggested [50].

The phylogenetic tree revealed three well-defined clades that identified and classified the representative isolates into three different subtypes. Overall, the clades identified in this analysis are in agreement with those defined in previous studies [7,11]. The rooted neighbor-joining (NJ) tree indicated that ST1 and ST2 share a common ancestor. The tree also revealed that ST3, ST4 and ST8, as well as ST6 and ST9 are closely related. Previous studies have also noted the close relationship between ST1 and ST2, as well as ST3 and ST4 [16,51]. 

*Blastocystis* ST1 was the highest prevalent (51.1%, 23/45) among the examined outpatients, followed by ST2 (24.4%, 11/45) and ST3 (17.8%, 8/45). In a recent study, the distribution of *Blastocystis* subtypes was investigated in three African countries by Alfellni et al. [52] including Libya, Nigeria and Liberia. Four subtypes were detected in the Libyan population with ST1 (50.0%, 19/38) as the highest prevalent followed by ST3 (39.5%, 15/38), ST2 (7.9%, 3/38) and ST7 (2.6%, 1/38). However, in this study, ST7 was not found which could be due to the differences in primers and subtyping method used by Alfellni et al. [52]. Distribution of *Blastocystis* sp. subtypes in the human population of several countries is listed in Table 4. It is shown that ST1 is widely reported in all listed countries and predominant in several countries including Libya, Thailand, Nigeria, Brazil and Iran. ST3 was predominant in most of the listed countries. ST2 occurred as the third most common except in Ireland and Tanzania. ST4 was the predominant subtype in Spain and commonly found in Denmark and UK. ST6, ST7, ST8 and ST9 were occasionally detected in several countries. These data show that the subtypes present in humans may vary with the geographic distribution of *Blastocystis* sp. and reflect differences in epidemiological characteristics, study population, reservoirs and dynamics of transmission [13,15]. 

**Table 4:** Subtype distribution of human *Blastocystis* sp. isolates from different countries.

Country (Ref)	Participant (no. of sample)	Subtype distribution									
		ST1	ST2	ST3	ST4	ST5	ST6	ST7	ST8	ST9	Unk/MSI
Libya*	*Symptomatic (29)*	*15*	*5*	*8*	*-*	*-*	*-*	*-*	*-*	*-*	*1*
	*Asymptomatic (16)*	*8*	*6*	*-*	*-*	*-*	*-*	*-*	*-*	*-*	*2*
Libya [52]	Outpatient (38)	19	3	15	-	-	-	1	-	-	-
Australia [72]	Patients (91)	28	5	40	12	-	3	1	2	-	-
Bangladesh [55]	*Symptomatic (11)*	*1*	*-*	*10*	*-*	*-*	*-*	*-*	*-*	*-*	*-*
	*Asymptomatic (15)*	*1*	*-*	*14*	*-*	*-*	*-*	*-*	*-*	*-*	*-*
Brazil [73]	Indigenous community (66)	27	21	11	-	-	-	-	-	-	7
China [23]	*Symptomatic (16)*	*9*	*-*	*2*	*-*	*-*	*-*	*-*	*-*	*-*	*5*
	*Asymptomatic (19)*	*4*	*-*	*12*	*-*	*-*	*-*	*2*	*-*	*-*	*1*
China [60]	Rural community (78)	16	1	55	1	-	-	-	-	-	5
Denmark [29]	General population (24)	2	3	6	9	-	-	-	-	1	3
	Patients (92)	19	19	15	11	-	-	5	-	-	23
Denmark [74]	Patients (99)	20	15	39	16	-	1	-	1	-	7
Egypt [67]	*Symptomatic (28)*	*8*	*-*	*16*	*-*	*-*	*4*	*-*	*-*	*-*	*-*
	*Asymptomatic (16)*	*-*	*-*	*8*	*-*	*-*	*4*	*4*	*-*	*-*	*-*
Egypt [75]	IBS patients (51)	15	-	22	-	-	8	-	-	-	6
	Control (49)	-	-	17	-	-	15	13	-	-	4
France [13]	*Symptomatic (25)*	*4*	*4*	*13*	*2*	*-*	*-*	*-*	*-*	*-*	*2*
	*Asymptomatic (15)*	*4*	*-*	*7*	*2*	*-*	*-*	*1*	*-*	*-*	*1*
France [76]	HM patients (15)	-	-	3	10	-	1	1	-	-	-
	Control (12)	1	1	1	7	-	-	2	-	-	-
Germany [55]	Patient (12)	3	2	5	2	-	-	-	-	-	-
Greece [77]	*Symptomatic (19)*	*-*	*-*	*18*	*1*	*-*	*-*	*-*	*-*	*-*	*-*
	*Asymptomatic (32)*	*7*	*5*	*14*	*-*	*-*	*1*	*5*	*-*	*-*	*-*
Iran [78]	Patient (45)	20	4	16	-	-	2	3	-	-	-
Ireland [79]	Healthy persons (14)	1	6	4	3	-	-	-	-	-	-
Italy [14]	Symptomatic (34)	3	7	16	6	-	-	1	1	-	-
Japan [55]	Patient (50)	4	0	26	2	0	11	5	0	2	0
Lebanon [80]	*Symptomatic (19)*	10	5	3	-	-	-	-	-	-	-
	*Asymptomatic (18)*	1	7	9	1	-	-	-	-	-	-
Liberia [52]	Schoolchildren (30)	7	7	8	3	-	-	-	-	-	5
Malaysia [81]	HIV patient (20)	3	1	11	5	-	-	-	-	-	-
	Cancer patient (20)	2	1	9	6	-	-	-	-	-	2
Mexico [30]	IBS patients (14)	2	1	2	-	-	-	-	-	-	9
	Control (6)	-	-	3	-	-	-	-	-	-	3
Nepal [10]	Symptomatic (20)	4	4	12	-	-	-	-	-	-	-
Nigeria [52]	Patient (23)	10	-	9	3	-	-	-	-	-	1
Pakistan [55]	Symptomatic (10)	2	-	7	-	-	1	-	-	-	-
Pakistan [82]	IBS patients (123)	75	6	23	6	3	3	5	-	-	2
	Control (56)	12	4	26	2	4	3	5	-	-	-
Singapore [54]	Patient (9)	2	-	7	-	-	-	-	-	-	-
Spain [22]	Symptomatic (51)	1	2	-	48	-	-	-	-	-	-
Sweden [83]	Patient (63)	10	9	30	13	-	-	1	-	-	-
Thailand [84]	Army personnel (153)	138	-	7	-	-	-	2	-	-	6
Thailand [85]	Schoolchildren (68)	53	15	-	-	-	-	-	-	-	-
Tanzania [86]	Healthy persons (6)	1	3	2	-	-	-	-	-	-	-
Turkey [12]	*Symptomatic (69)*	*6*	*9*	*53*	*1*	*-*	*-*	*-*	*-*	*-*	*-*
	*Asymptomatic (18)*	*2*	*3*	*13*	*-*	*-*	*-*	*-*	*-*	*-*	*-*
UK [47]	Patient (54)	3	9	22	17	-	-	-	3	-	-
UK [52]	IBS patients (136)	14	10	56	51	-	1	1	3	-	-
	Control (135)	20	16	58	34	2	-	3	2	-	-
USA [65]	Symptomatic (9)	1	-	6	-	-	-	-	-	-	2
Total sample	2141	618	219	789	274	9	58	61	12	3	97

Current study (*), mixed subtype infection (MSI), unknown (Unk), irritable bowel syndrome (IBS), hematological malignancies (HM), symptomatic (with gastrointestinal symptoms), asymptomatic (without gastrointestinal symptoms).

ST3 was predominant in humans [2,12,54,55] but relatively low prevalent in different animals [16,19,41,53]. It was suggested to be the only subtype of human origin and has human-to-human transmission [2,54,55]. The other remaining subtypes were likely of zoonotic origin [2,7,55,56]. Additionally, ST1 and ST2 support the low host specificity found in a wide range of animals including monkeys, chickens, cattle, pigs, dogs and non-human primates [7,57,58]. In the current study, ST1 and ST2 were found to be common in Libyan outpatients coming from varied family backgrounds, such as agricultural, animal husbandry, private and government workers. Most families lived nearby Sebha city and owned animal farms surrounding their houses. Therefore, the occurrence of zoonotic transmission of *Blastocystis* in this population is a possibility. However, in a recent study, *Blastocystis* ST1 and ST3 but not ST2 were isolated from camels and goats in Libya at relatively low percentages (ST1 7%, ST3 9%) [19]. The same subtypes were previously found to be dominant (ST1 50%, ST3 39.5%) in Libyan population [52] suggesting that zoonotic transmission is unlikely to occur [19]. However, the animals with which the Libyan population has the most contact are sheep and these have not been sampled in Libya [19]. Therefore, more studies on *Blastocystis* in animals in Libya particularly those in close contact with humans are required. 

The majority (93.3%, 42/45) had a single subtype infection whereas 6.7% (3/45) had mixed infections with two concurrent subtypes. The presence of multiple subtypes may indicate the concurrent existence of different sources of infection or one source containing multiple subtypes. Besides, the existence of mixed infections observed in this study may be underestimated since only three to four clones were sequenced per sample. Recently, sequencing up to ten clones [41], enabled three different subtypes in a primate isolate to be identified. Furthermore, utilizing *in vitro* culture prior to PCR can also be a source of underestimation of mixed infections, as this method may favour the preferential growth of a certain subtype over the others [9,59]. Hence, genotyping of *Blastocystis* DNA obtained directly from stools may be more accurate for identifying mixed infections if PCR conditions are optimal [2]. In general, the prevalence of mixed infections in our study is similar to that described in different countries, which ranged between 2.6% and 14.3% [13,14,29,60,61]. In the latter conditions, mixed infections were expected with several concurrent subtypes such as ST1 and ST3, ST1 and ST2, or ST2 and ST3.

The present study showed differences in the occurrence of *Blastocystis* ST1 infection between male and female outpatients. This finding contradicts previous studies which found no significant associations between gender and subtypes of *Blastocystis* sp. [12,25,62]. Differences in the relative proportions of subtypes between the genders could indicate distinct ways of transmission or different exposures to the sources of transmission [60]. As our study involved a small sample size, further identification in a greater number of subtypes is needed to confirm the association between *Blastocystis* subtypes against the demographic and socioeconomic characteristics of the outpatients. Our findings showed that ST2 was significantly associated with low level of education. It is well documented that the level of education can influence the prevalence of parasitic infections including *Blastocystis* sp. [36,63], and that hygienic conditions and sanitary practices would be better in those with a higher level of education.

The pathogenic potential of *Blastocystis* remains uncertain because of the conflicting reports about clinical symptoms caused by *Blastocystis* infection. Recently, it has been generally assumed that certain subtypes may contribute to the pathogenicity. In this study, all patients with ST3 presented with gastrointestinal symptoms and had no other pathogenic parasite infections. Fisher’s exact test showed that ST3 was significantly related with the presence of diarrhoea. This finding is in agreement with previous reports which stated that ST3 was predominant in patients with chronic gastrointestinal illness in several countries including Malaysia [64], Singapore [54], the USA [65] and Egypt [61]. ST3 infection was significantly higher in symptomatic Thai patients (especially diarrhoea) as compared to asymptomatic individuals [66]. In our study, despite the suggestion of an association between ST3 and its possible pathogenicity, our sample size was too small and investigation on the presence of virus or bacteria was not conducted.

In the current study, ST1 infection is more common among symptomatic individuals compared to asymptomatic individuals. However, no significant association was found between *Blastocystis* ST1 infection and gastrointestinal symptoms. One symptomatic patient (L18-9/F) with ST1 (isolate LFS-4) had *Giardia intestinalis* co-infection, which could have been the main cause of intestinal symptoms. Previous studies have demonstrated that ST1 was associated with elevated pathogenicity [21,23,67]. However, a report by Tan et al [64] indicated otherwise; ST1 was only detected among the isolates from asymptomatic groups. As for ST2, 6 and 5 isolates were detected in asymptomatic and symptomatic patients, respectively. An asymptomatic patient (L5-27/M) was found to be co-infected with *Cryptosporidium* species. On the other hand, ST2 has been suggested to be a non-pathogenic genotype of *Blastocystis* [21,25] although several reports found that ST2 was associated with symptomatic infections [29,68]. The correlation between *Blastocystis* incidence and symptoms and the lack of correlation between specific subtypes and symptoms may be due to the fact that the clinical outcome of *Blastocystis* infection is multifactorial, involving host factors (genetics, immunity and age) or parasite factors (genotype, virulence and zoonotic potential) or a combination of the two [69]. As a limitation, this study may a selection bias as the samples were collected from patients presented to the Sebha Central Laboratory. Moreover, the patients were not keen to bring subsequent stool sample, and a single stool sample collection could influence the detection rate of *Blastocystis* and other intestinal parasites. For optimum parasite detection, at least three specimens collected on three successive days is recommended. 

In conclusion, this is the first molecular study on the associated factors of human *Blastocystis* infection in Libya. The genetic polymorphism of SSU rDNA among the different *Blastocystis* isolates found in this study showed that these organisms are genetically highly divergent. Among the three identified subtypes, ST1 was predominant and its infection was significantly associated with female gender and low level of education. Although all three subtypes were detected in symptomatic outpatients, ST3 infection was found to be significantly associated with gastrointestinal symptoms, indicating that the pathogenic potential of *Blastocystis* sp. might be subtype-related. Despite the suggestion of an association between ST3 and its possible pathogenicity, our sample size was too small and detection of virus or bacteria was not conducted, and thus further studies are needed to confirm this issue. 

## Supporting Information

Table S1 **General characteristics of the outpatients (n = 380) against *Blastocystis* infection.**
(DOCX)

Table S2 **Patient record and percentage homology of infected *Blastocystis* subtypes with their closest match reference from Genbank.**
(PDF)

## References

[1] Silberman JD, Sogin ML, Leipe DD, Clark CG (1996) Human parasite finds taxonomic home. Nature 380: 398-398. doi:10.1038/380398a0. PubMed: 8602239. 8602239

[2] Tan KSW (2008) New Insights on Classification, Identification, and Clinical Relevance of *Blastocystis* spp. Clin Microbiol Rev 21: 639-665. doi:10.1128/cmr.00022-08. PubMed: 18854485. 18854485 PMC2570156

[3] Sohail MR, Fischer PR (2005) *Blastocystis* *hominis* and travelers. Travel Med Infect Dis 3: 33-38. doi:10.1016/j.tmaid.2004.06.001. PubMed: 17292002. 17292002

[4] Lee LI, Chye TT, Karmacharya BM, Govind SK (2012) *Blastocystis* sp.: Waterborne zoonotic organism, a possibility? Parasit Vectors 5: 130. doi:10.1186/1756-3305-5-130. PubMed: 22741573. 22741573 PMC3434050

[5] Abdulsalam AM, Ithoi I, Al-Mekhlafi HM, Ahmed A, Surin J et al. (2012) Drinking water is a significant predictor of *Blastocystis* infection among rural Malaysian primary schoolchildren. Parasitology 139: 1014-1020. doi:10.1017/s0031182012000340. PubMed: 22444778. 22444778

[6] Yoshikawa H, Yoshida K, Nakajima A, Yamanari K, Iwatani S et al. (2004) Fecal-oral transmission of the cyst form of *Blastocystis* *hominis* in rats. Parasitol Res 94: 391-396. doi:10.1007/s00436-004-1230-5. PubMed: 15480786. 15480786

[7] Noël C, Dufernez F, Gerbod D, Edgcomb VP, Delgado-Viscogliosi P et al. (2005) Molecular phylogenies of *Blastocystis* isolates from different hosts: implications for genetic diversity, identification of species, and zoonosis. J Clin Microbiol 43: 348-355. doi:10.1128/JCM.43.1.348-355.2005. PubMed: 15634993. 15634993 PMC540115

[8] Parkar U, Traub RJ, Vitali S, Elliot A, Levecke B et al. (2010) Molecular characterization of *Blastocystis* isolates from zoo animals and their animal-keepers. Vet Parasitol 169: 8-17. doi:10.1016/j.vetpar.2009.12.032. PubMed: 20089360. 20089360

[9] Yan Y, Su S, Ye J, Lai X, Lai R et al. (2007) *Blastocystis* sp. subtype 5: a possibly zoonotic genotype. Parasitol Res 101: 1527-1532. doi:10.1007/s00436-007-0672-y. PubMed: 17665214. 17665214

[10] Yoshikawa H, Wu Z, Pandey K, Pandey BD, Sherchand JB et al. (2009) Molecular characterization of *Blastocystis* isolates from children and rhesus monkeys in Kathmandu, Nepal. Vet Parasitol 160: 295-300. doi:10.1016/j.vetpar.2008.11.029. PubMed: 19136214. 19136214

[11] Stensvold CR, Suresh GK, Tan KSW, Thompson RCA, Traub RJ et al. (2007) Terminology for *Blastocystis* subtypes – a consensus. Trends Parasitol 23: 93-96. doi:10.1016/j.pt.2007.01.004. PubMed: 17241816. 17241816

[12] Ozyurt M, Kurt Ö, Mølbak K, Nielsen HV, Haznedaroglu T et al. (2008) Molecular epidemiology of *Blastocystis* infections in Turkey. Parasitol Int 57: 300-306. doi:10.1016/j.parint.2008.01.004. PubMed: 18337161. 18337161

[13] Souppart L, Sanciu G, Cian A, Wawrzyniak I, Delbac F et al. (2009) Molecular epidemiology of human *Blastocystis* isolates in France. Parasitol Res 105: 413-421. doi:10.1007/s00436-009-1398-9. PubMed: 19290540. 19290540

[14] Meloni D, Sanciu G, Poirier P, Alaoui H, Chabé M et al. (2011) Molecular subtyping of *Blastocystis* sp. isolates from symptomatic patients in Italy. Parasitol Res 109: 613-619. doi:10.1007/s00436-011-2294-7. PubMed: 21340563. 21340563

[15] Li LH, Zhang XP, Lv S, Zhang L, Yoshikawa H et al. (2007) Cross-sectional surveys and subtype classification of human *Blastocystis* isolates from four epidemiological settings in China. Parasitol Res 102: 83-90. doi:10.1007/s00436-007-0727-0. PubMed: 17912552. 17912552

[16] Stensvold CR, Alfellani MA, Nørskov-Lauritsen S, Prip K, Victory EL et al. (2009) Subtype distribution of *Blastocystis* isolates from synanthropic and zoo animals and identification of a new subtype. Int J Parasitol 39: 473-479. doi:10.1016/j.ijpara.2008.07.006. PubMed: 18755193. 18755193

[17] Fayer R, Santin M, MacArisin D (2012) Detection of concurrent infection of dairy cattle with *Blastocystis*, *Cryptosporidium*, *Giardia*, and *Enterocytozoon* by molecular and microscopic methods. Parasitol Res 111: 1349-1355. doi:10.1007/s00436-012-2971-1. PubMed: 22710524. 22710524

[18] Roberts T, Stark D, Harkness J, Ellis J (2013) Subtype distribution of *Blastocystis* isolates from a variety of animals from New South Wales, Australia. Vet Parasitol 196: 85-89. doi:10.1016/j.vetpar.2013.01.011. PubMed: 23398989. 23398989

[19] Alfellani MA, Taner-Mulla D, Jacob AS, Imeede CA, Yoshikawa H et al. (2013) Genetic Diversity of *Blastocystis* in Livestock and Zoo Animals. Protist 164: 497-509. doi:10.1016/j.protis.2013.05.003. PubMed: 23770574. 23770574

[20] Tan KS, Mirza H, Teo JD, Wu B, MacAry PA (2010) Current Views on the Clinical Relevance of *Blastocystis* spp. Curr Infect Dis Rep 12: 28-35. doi:10.1007/s11908-009-0073-8. PubMed: 21308496. 21308496

[21] Eroglu F, Genc A, Elgun G, Koltas IS (2009) Identification of *Blastocystis* *hominis* isolates from asymptomatic and symptomatic patients by PCR. Parasitol Res 105: 1589-1592. doi:10.1007/s00436-009-1595-6. PubMed: 19685075. 19685075

[22] Domínguez-Márquez MV, Guna R, Muñoz C, Gómez-Muñoz MT, Borrás R (2009) High prevalence of subtype 4 among isolates of *Blastocystis* *hominis* from symptomatic patients of a health district of Valencia (Spain). Parasitol Res 105: 949-955. doi:10.1007/s00436-009-1485-y. PubMed: 19471964. 19471964

[23] Yan Y, Su S, Lai R, Liao H, Ye J et al. (2006) Genetic variability of *Blastocystis* *hominis* isolates in China. Parasitol Res 99: 597-601. doi:10.1007/s00436-006-0186-z. PubMed: 16688468. 16688468

[24] Tan TC, Suresh KG (2006) Amoeboid form of *Blastocystis* *hominis*—a detailed ultrastructural insight. Parasitol Res 99: 737-742. doi:10.1007/s00436-006-0214-z. PubMed: 16816959. 16816959

[25] Dogruman-Al F, Dagci H, Yoshikawa H, Kurt Ö, Demirel M (2008) A possible link between subtype 2 and asymptomatic infections of *Blastocystis* *hominis* . Parasitol Res 103: 685-689. doi:10.1007/s00436-008-1031-3. PubMed: 18523804. 18523804

[26] Kaya S, Cetin ES, Aridoğan B, Arikan S, Demirci M (2007) Pathogenicity of *Blastocystis* *hominis*, a clinical reevaluation. Türkiye Parazitolojii Dergisi 31: 184-187. PubMed: 17918055. 17918055

[27] Qadri SM, al-Okaili GA, al-Dayel F (1989) Clinical significance of *Blastocystis* *hominis* . J Clin Microbiol 27: 2407-2409. PubMed: 2808664. 2808664 10.1128/jcm.27.11.2407-2409.1989 PMC267045

[28] Yakoob J, Jafri W, Jafri N, Khan R, Islam M et al. (2004) Irritable bowel syndrome: in search of an etiology: role of *Blastocystis* *hominis* . Am J Trop Med Hyg 70: 383-385. PubMed: 15100450. 15100450

[29] Stensvold CR, Lewis HC, Hammerum AM, Porsbo LJ, Nielsen SS et al. (2009) *Blastocystis*: unravelling potential risk factors and clinical significance of a common but neglected parasite. Epidemiol Infect 137: 1655-1663. doi:10.1017/s0950268809002672. PubMed: 19393117. 19393117

[30] Jimenez-Gonzalez DE, Martinez-Flores WA, Reyes-Gordillo J, Ramirez-Miranda ME, Arroyo-Escalante S et al. (2012) *Blastocystis* infection is associated with irritable bowel syndrome in a Mexican patient population. Parasitol Res 110: 1269-1275. doi:10.1007/s00436-011-2626-7. PubMed: 21870243. 21870243

[31] Boorom KF, Smith H, Nimri L, Viscogliosi E, Spanakos G et al. (2008) Oh my aching gut: irritable bowel syndrome, *Blastocystis*, and asymptomatic infection. Parasit Vectors 1: 1-16. doi:10.1186/1756-3305-1-40. PubMed: 18272002. 18937874 PMC2627840

[32] Al-Fellani MA, Khan AH, Al-Gazoui RM, Zaid MK, Al-Ferjani MA (2007) Prevalence and clinical features of *Blastocystis* *hominis* infection among patients in Sebha, Libya. Sultan Qaboos Univ Med J 7: 35-40. PubMed: 21654943. 21654943 PMC3086416

[33] Kasssem HH, Zaed HA, Sadaga GA (2007) Intestinal parasitic infection among children and neonatus admitted to Ibn-Sina Hospital, Sirt, Libya. J Egypt Soc Parasitol 37: 371-380. PubMed: 17985574. 17985574

[34] Sadaga GA, Kassem HH (2007) Prevalence of intestinal parasites among primary schoolchildren in Derna District, Libya. J Egypt Soc Parasitol 37: 205-214. PubMed: 17580578. 17580578

[35] Salem RAA, Abdullah ME, Abdulgader AE (2006) Intestinal protozoa in Libyan patients in Sirt. Jamahiriya Medical Journal 6: 59-61.

[36] Abdulsalam AM, Ithoi I, Al-Mekhlafi HM, Khan AH, Ahmed A et al. (2013) Prevalence, predictors and clinical significance of *Blastocystis* sp. in Sebha, Libya. Parasit Vectors 6: 86. doi:10.1186/1756-3305-6-86. PubMed: 23566585Available: . doi:10.1186/1756-3305-6-86. 23566585 PMC3626707

[37] Leelayoova S, Taamasri P, Rangsin R, Naaglor T, Thathaisong U et al. (2002) In-vitro cultivation: a sensitive method for detecting *Blastocystis* *hominis* . Ann Trop Med Parasitol 96: 803-807. doi:10.1179/000349802125002275. PubMed: 12625935. 12625935

[38] Zhang X, Qiao J, Wu X, Da R, Zhao L et al. (2012) In vitro culture of *Blastocystis* *hominis* in three liquid media and its usefulness in the diagnosis of blastocystosis. Int J Infect Dis 16: e23-e28. doi:10.1016/j.ijid.2011.09.012. PubMed: 22047715. 22047715

[39] Zman V, Khan KZ (1994) A comparison of direct microscopy with culture for the diagnosis of *Blastocystis* *hominis* . Southeast Asian J Trop Med Public Health 25: 792-793. PubMed: 7667737. 7667737

[40] Böhm-Gloning B, Knobloch J, Walderich B (1997) Five subgroups of *Blastocystis* *hominis* isolated from symptomatic and asymptomatic patients revealed by restriction site analysis of PCR-amplified 16S-like rDNA. Trop Med Int Health 2: 771-778. doi:10.1046/j.1365-3156.1997.d01-383.x. PubMed: 9294547. 9294547

[41] Santín M, Gómez-Muñoz MT, Solano-Aguilar G, Fayer R (2011) Development of a new PCR protocol to detect and subtype *Blastocystis* spp. from humans and animals. Parasitol Res 109: 205-212. doi:10.1007/s00436-010-2244-9. PubMed: 21210149. 21210149

[42] Hall TA (1999) BioEdit: a user-friendly biological sequence alignment editor and analysis program for Windows 95/98/NT. Nucleic Acids Symp Ser 41: 95-98.

[43] Tamura K, Dudley J, Nei M, Kumar S (2007) MEGA4: Molecular Evolutionary Genetics Analysis (MEGA) software version 4.0. Mol Biol Evol 24: 1596-1599. doi:10.1093/molbev/msm092. PubMed: 17488738. 17488738

[44] Kimura M (1980) simple method for estimating evolutionary rates of base substitutions through comparative studies of nucleotide sequences. J Mol Evol 16: 111-120. doi:10.1007/BF01731581. PubMed: 7463489. 7463489

[45] Stensvold R, Brillowska-Dabrowska A, Nielsen HV, Arendrup MC (2006) Detection of *Blastocystis* *hominis* in unpreserved stool specimens by using Polymerase Chain Reaction. J Parasitol 92: 1081-1087. doi:10.1645/ge-840r.1. PubMed: 17152954. 17152954

[46] Stensvold CR, Nielsen HV, Mølbak K, Smith HV (2009) Pursuing the clinical significance of *Blastocystis* – diagnostic limitations. Trends Parasitol 25: 23-29. doi:10.1016/j.pt.2008.09.010. PubMed: 19013108. 19013108

[47] Scicluna SM, Tawari B, Clark CG (2006) DNA Barcoding of *Blastocystis* . Protist 157: 77-85. doi:10.1016/j.protis.2005.12.001. PubMed: 16431158. 16431158

[48] Arisue N, Hashimoto T, Yoshikawa H (2003) Sequence heterogeneity of the small subunit ribosomal RNA genes among *Blastocystis* isolates. Parasitology 126: 1-9. doi:10.1017/s0031182002002640. PubMed: 12613758. 12613758

[49] Noël C, Peyronnet C, Gerbod D, Edgcomb VP, Delgado-Viscogliosi P et al. (2003) Phylogenetic analysis of *Blastocystis* isolates from different hosts based on the comparison of small-subunit rRNA gene sequences. Mol Biochem Parasitol 126: 119-123. doi:10.1016/s0166-6851(02)00246-3. PubMed: 12554093. 12554093

[50] Stensvold CR, Alfellani M, Clark CG (2012) Levels of genetic diversity vary dramatically between *Blastocystis* subtypes. Infect Genet Evol 12: 263-273. doi:10.1016/j.meegid.2011.11.002. PubMed: 22116021. 22116021

[51] Whipps CM, Boorom K, Bermudez LE, Kent ML (2010) Molecular characterization of *Blastocystis* species in Oregon identifies multiple subtypes. Parasitol Res 106: 827-832. doi:10.1007/s00436-010-1739-8. PubMed: 20127113. 20127113

[52] Alfellani MA, Stensvold CR, Vidal-Lapiedra A, Onuoha E, Fagbenro-Beyioku A et al. (2013) Variable geographic distribution of *Blastocystis* subtypes and its potential implications. Acta Trop 126: 11-18. doi:10.1016/j.actatropica.2012.12.011. PubMed: 23290980. 23290980

[53] Alfellani MA, Jacob AS, Perea NO, Krecek RC, Taner-Mulla D et al. (2013) Diversity and distribution of *Blastocystis* sp. subtypes in non-human primates. Parasitology 140: 966-971. doi:10.1017/S0031182013000255. PubMed: 23561720. 23561720

[54] Wong KHS, Ng GC, Lin RTP, Yoshikawa H, Taylor MB et al. (2008) Predominance of subtype 3 among *Blastocystis* isolates from a major hospital in Singapore. Parasitol Res 102: 663-670. doi:10.1007/s00436-007-0808-0. PubMed: 18064490. 18064490

[55] Yoshikawa H, Wu Z, Kimata I, Iseki M, Ali ID et al. (2004) Polymerase chain reaction-based genotype classification among human *Blastocystis* *hominis* populations isolated from different countries. Parasitol Res 92: 22-29. doi:10.1007/s00436-003-0995-2. PubMed: 14598169. 14598169

[56] Yoshikawa H, Abe N, Iwasawa M, Kitano S, Nagano I et al. (2000) Genomic Analysis of *Blastocystis* *hominis* strains Isolated from Two Long-Term Health Care Facilities. J Clin Microbiol 38: 1324-1330. PubMed: 10747102. 10747102 10.1128/jcm.38.4.1324-1330.2000 PMC86440

[57] Abe N, Wu Z, Yoshikawa H (2003) Molecular characterization of *Blastocystis* isolates from primates. Vet Parasitol 113: 321-325. doi:10.1016/s0304-4017(03)00081-5. PubMed: 12719144. 12719144

[58] Yoshikawa H, Abe N, Wu Z (2004) PCR-based identification of zoonotic isolates of *Blastocystis* from mammals and birds. Microbiology 150: 1147–1151. doi:10.1099/mic.0.26899-0. PubMed: 15133074. 15133074

[59] Parkar U, Traub RJ, Kumar S, Mungthin M, Vitali S et al. (2007) Direct characterization of *Blastocystis* from faeces by PCR and evidence of zoonotic potential. Parasitology 134: 359-367. doi:10.1017/s0031182006001582. PubMed: 17052374. 17052374

[60] Li L-H, Zhou X-N, Du Z-W, Wang X-Z, Wang L-B et al. (2007) Molecular epidemiology of human *Blastocystis* in a village in Yunnan province, China. Parasitol Int 56: 281-286. doi:10.1016/j.parint.2007.06.001. PubMed: 17627869. 17627869

[61] Souppart L, Moussa H, Cian A, Sanciu G, Poirier P et al. (2010) Subtype analysis of *Blastocystis* isolates from symptomatic patients in Egypt. Parasitol Res 106: 505-511. doi:10.1007/s00436-009-1693-5. PubMed: 19953268. 19953268

[62] Dogruman-Al F, Yoshikawa H, Kustimur S, Balaban N (2009) PCR-based subtyping of *Blastocystis* isolates from symptomatic and asymptomatic individuals in a major hospital in Ankara, Turkey. Parasitol Res 106: 263-268. doi:10.1007/s00436-009-1658-8. PubMed: 19847459. 19847459

[63] Aksoy U, Akisü C, Bayram-Delibaş S, Ozkoç S, Sahin S et al. (2007) Demographic status and prevalence of intestinal parasitic infections in schoolchildren in Iznir in Turkey. Turk J Pediatr 49: 278-282. PubMed: 17990581. 17990581

[64] Tan TC, Suresh KG, Smith HV (2008) Phenotypic and genotypic characterisation of *Blastocystis* *hominis* isolates implicates subtype 3 as a subtype with pathogenic potential. Parasitol Res 104: 85-93. doi:10.1007/s00436-008-1163-5. PubMed: 18795333. 18795333

[65] Jones MS, Whipps CM, Ganac RD, Hudson NR, Boorom K (2009) Association of *Blastocystis* subtype 3 and 1 with patients from an Oregon community presenting with chronic gastrointestinal illness. Parasitol Res 104: 341-345. doi:10.1007/s00436-008-1198-7. PubMed: 18923844. 18923844

[66] Jantermtor S, Pinlaor P, Sawadpanich K, Pinlaor S, Sangka A et al. (2013) Subtype identification of *Blastocystis* spp. isolated from patients in a major hospital in northeastern Thailand. Parasitol Res 112: 1781-1786. doi:10.1007/s00436-012-3218-x. PubMed: 23224731. 23224731

[67] Hussein EM, Hussein AM, Eida MM, Atwa MM (2008) Pathophysiological variability of different genotypes of human *Blastocystis* *hominis* Egyptian isolates in experimentally infected rats. Parasitol Res 102: 853-860. doi:10.1007/s00436-007-0833-z. PubMed: 18193282. 18193282

[68] Vogelberg C, Stensvold CR, Monecke S, Ditzen A, Stopsack K et al. (2010) *Blastocystis* sp. subtype 2 detection during recurrence of gastrointestinal and urticarial symptoms. Parasitol Int 59: 469-471. doi:10.1016/j.parint.2010.03.009. PubMed: 20363362. 20363362

[69] Scanlan PD (2012) *Blastocystis*: Past pitfalls and future perspectives. Trends Parasitol 28: 327-334. doi:10.1016/j.pt.2012.05.001. PubMed: 22738855. 22738855

[70] Abe N (2004) Molecular and phylogenetic analysis of *Blastocystis* isolates from various hosts. Vet Parasitol 120: 235-242. doi:10.1016/j.vetpar.2004.01.003. PubMed: 15041098. 15041098

[71] Rivera WL (2008) Phylogenetic analysis of *Blastocystis* isolates from animal and human hosts in the Philippines. Vet Parasitol 156: 178-182. doi:10.1016/j.vetpar.2008.06.001. PubMed: 18606497. 18606497

[72] Roberts T, Stark D, Harkness J, Ellis J (2013) Subtype distribution of *Blastocystis* isolates identified in a Sydney population and pathogenic potential of *Blastocystis* . Eur J Clin Microbiol Infect Dis 32: 335-343. doi:10.1007/s10096-012-1746-z. PubMed: 22996007. 22996007

[73] Malheiros AF, Stensvold CR, Clark CG, Braga GB, Shaw JJ (2011) Short report: Molecular characterization of *Blastocystis* obtained from members of the indigenous tapirapé ethnic group from the Brazilian Amazon Region, Brazil. Am J Trop Med Hyg 85: 1050-1053. doi:10.4269/ajtmh.2011.11-0481. PubMed: 22144442. 22144442 PMC3225150

[74] Rene BA, Stensvold CR, Badsberg JH, Nielsen HV (2009) Subtype analysis of *Blastocystis* isolates from *Blastocystis* cyst excreting patients. Am J Trop Med Hyg 80: 588-592. PubMed: 19346381. 19346381

[75] Fouad SA, Basyoni MMA, Fahmy RA, Kobaisi MH (2011) The pathogenic role of different *Blastocystis* *hominis* genotypes isolated from patients with irritable bowel syndrome. Arab. Journal of Gastroenterology 12: 194-200. doi:10.1016/j.ajg.2011.11.005. 22305500

[76] Poirier P, Wawrzyniak I, Al Albert, El Alaoui H, Delbac F et al. (2011) Development and evaluation of a Real-Time PCR assay for detection and quantification of *Blastocystis* parasites in human stool samples: prospective study of patients with hematological malignancies. J Clin Microbiol 49: 975–983. doi:10.1128/JCM.01392-10. PubMed: 21177897. 21177897 PMC3067686

[77] Vassalos CM, Spanakos G, Vassalou E, Papadopoulou C, Vakalis N (2010) Differences in clinical significance and morphologic features of *Blastocystis* sp subtype 3. Am J Clin Pathol 133: 251-258. doi:10.1309/AJCPDOWQSL6E8DMN. PubMed: 20093234. 20093234

[78] Motazedian H, Ghasemi H, Sadjjadi SM (2008) Genomic diversity of *Blastocystis* *hominis* in southern Iran. Ann Trop Med Parasitol 102: 85-88. doi:10.1179/136485908X252197. PubMed: 18186983. 18186983

[79] Scanlan PD, Marchesi JR (2008) Micro-eukaryotic diversity of the human distal gut microbiota: qualitative assessment using culture-dependent and independent analysis of faeces. ISME J 2: 1183-1193. doi:10.1038/ismej.2008.76. PubMed: 18670396. 18670396

[80] El Safadi D, Meloni D, Poirier P, Osman M, Cian A et al. (2013) Molecular epidemiology of *Blastocystis* in Lebanon and correlation between subtype 1 and gastrointestinal symptoms. Am J Trop Med Hyg, 88: 1203–6. doi:10.4269/ajtmh.12-0777. PubMed: 23458955. 23458955 PMC3752823

[81] Tan TC, Ong SC, Suresh KG (2009) Genetic variability of *Blastocystis* sp. isolates obtained from cancer and HIV/AIDS patients. Parasitol Res 105: 1283-1286. doi:10.1007/s00436-009-1551-5. PubMed: 19603182. 19603182

[82] Yakoob J, Jafri W, Beg MA, Abbas Z, Naz S et al. (2010) Irritable bowel syndrome: is it associated with genotypes of *Blastocystis* *hominis* . Parasitol Res 106: 1033-1038. doi:10.1007/s00436-010-1761-x. PubMed: 20177906. 20177906

[83] Forsell J, Granlund M, Stensvold CR, Clark GC, Evengård B (2012) Subtype analysis of *Blastocystis* isolates in Swedish patients. Eur J Clin Microbiol Infect Dis 31: 1689-1696. doi:10.1007/s10096-011-1416-6. PubMed: 22350386. 22350386

[84] Thathaisong U, Worapong J, Mungthin M, Tan-Ariya P, Viputtigul K et al. (2003) *Blastocystis* isolates from a pig and a horse are closely related to *Blastocystis* *hominis* . J Clin Microbiol 41: 967-975. doi:10.1128/JCM.41.3.967-975.2003. PubMed: 12624017. 12624017 PMC150316

[85] Leelayoova S, Siripattanapipong S, Thathaisong U, Naaglor T, Taamasri P et al. (2008) Drinking Water: A Possible Source of *Blastocystis* spp. Subtype 1 Infection in Schoolchildren of a Rural Community in Central Thailand. Am J Trop Med Hyg 79: 401-406. PubMed: 18784233. 18784233

[86] Petrášová J, Uzlíková M, Kostka M, Petrželková KJ, Huffman MA et al. (2011) Diversity and host specificity of *Blastocystis* in syntopic primates on Rubondo Island, Tanzania. Int J Parasitol 41: 1113-1120. doi:10.1016/j.ijpara.2011.06.010. PubMed: 21854778. 21854778

