# Supplementary material for: Subtype Distribution of Blastocystis Isolates in Sebha, Libya
Source: PLoS One. 2013 Dec 20;8(12):e84372. doi: 10.1371/journal.pone.0084372 (PMC3869855; doi:10.1371/journal.pone.0084372)
Supplement: Table S1 — General characteristics of the outpatients (n = 380) against Blastocystis infection. (DOCX) [file pone.0084372.s001.docx]

| Variable | | Total | *Blastocystis* positive n (%) | *Blastocystis* negative n (%) |
| --- | --- | --- | --- | --- |
| Age | ≥18 years | 238 | 70 (29.4) | 168 (70.6) |
|  | <18 years | 142 | 14 (9.9) | 128 (90.1) |
| Gender | Male | 197 | 52 (26.4) | 145 (73.6) |
|  | Female | 183 | 32 (17.5) | 151 (82.5) |
| Education level | Low (≤ Primary school) | 193 | 51 (26.4) | 142 (73.6) |
|  | High (≥ Secondary school ) | 187 | 33 (17.6) | 154 (82.6) |
| Occupational status | Working | 132 | 42 (31.8) | 90 (68.2) |
|  | Not working | 168 | 33 (19.6) | 135 (80.4) |
| Family size | ≥ 7 members (large) | 222 | 40 (18.0) | 182 (82.0) |
|  | < 7 members | 158 | 44 (27.8) | 114 (72.2) |
| Drinking water | Untreated water | 242 | 56 (23.1) | 186 (76.9) |
|  | Treated water (chlorinated, filtered or boiled) | 138 | 28 (20.3) | 110 (79.7) |
| Presence of animals | Yes | 44 | 6 (13.6) | 38 (86.4) |
| in the house | No | 336 | 78 (23.3) | 258 (76.7) |
| History of recent | Yes | 16 | 5 (31.3) | 11 (68.7) |
| overseas travel | No | 364 | 79 (21.7) | 285 (78.3) |
| Presence of GI symptoms | Yes | 153 | 54 (35.3) | 99 (64.7) |
|  | No | 227 | 30 (13.2) | 97 (86.8) |
| Abdominal pain | Yes | 131 | 47 (35.9) | 84 (64.1) |
|  | No | 249 | 37 (14.9) | 212 (85.1) |
| Diarrhoea | Yes | 41 | 13 (31.7) | 28 (68.3) |
|  | No | 339 | 71 (20.9) | 268 (79.1) |
| Flatulence | Yes | 55 | 27 (49.1) | 28 (50.9) |
|  | No | 325 | 57 (17.5) | 268 (82.5) |
| Nausea/ vomiting | Yes | 5 | 2 (40.0) | 3 (60.0) |
|  | No | 375 | 82 (21.9) | 293 (78.1) |
| Constipation | Yes | 34 | 6 (17.6) | 28 (82.4) |
|  | No | 346 | 78 (22.5) | 268 (77.5) |

Table S1. General characteristics of the outpatients (n = 380) against *Blastocystis* infection.
