# Supplementary material for: Subtype Distribution of Blastocystis Isolates in Sebha, Libya
Source: PLoS One. 2013 Dec 20;8(12):e84372. doi: 10.1371/journal.pone.0084372 (PMC3869855; doi:10.1371/journal.pone.0084372)
Supplement: Table S2 — Patient record and percentage homology of infected Blastocystis subtypes with their closest match reference from Genbank. (PDF) [file pone.0084372.s002.pdf]

**Table S2.** Patient record and percentage homology of infected *Blastocystis* subtypes with their closest match reference from Genbank

| Patient no. - age/ gender | *Blastocystis* from symptomatic patient | | | | Genbank reference *Blastocystis* isolate, host (Accession no.) | | | *Blastocystis* from asymptomatic patient | | Patient no. - age/ gender |
|---|---|---|---|---|---|---|---|---|---|---|
| | Isolate designation | % homology | ST | Clones sequences with a single *Blastocystis* subtype<br>clone | | clone | ST | %homology | Isolate designation | |
| L1-15/F | **LFS-1** | (100) | | **(a,b,c)** | HJ96A-29, human (AB070989) | (a,b,c) | | (100) | LF-30 | L20-23/F |
| L16-38/F | LFS-2 | (100) | | (a,b,c) | MJ99-424, monkey (AB107967) | (a,b,c) | | (100) | LM-31 | L28-13/M |
| L17-60/F | LFS-3 | (100) | | (a,b,c) | | (a,b,c) | | (100) | LF-32 | L25-43/F |
| L18-9/F | LFS-4 | (100) | | (a,b,c) | | (a,b) **(c,d)** | | (100) **(99.7)** | **LM-33** | L22-75/M |
| L12-25/M | **LMS-5** | (100) **(99.7)** | | (a,b) **c,d)** | | (a,b,c) | | (100) | LM-34 | L24-50/M |
| L13-45/F | **LFS-6** | **(98.7)** (98.5) | | **(a,b)** c,d) | | (a,c) **(b,d)** | | (98.7) **(98.5)** | **LM-35** | L26-28/M |
| L31-9/M | LMS-7 | (100) | | (a,b,c) | | (a,b,c) | | (100) | LF-36 | L40-43/F |
| L32-5/M | LMS-8 | (100) | ST1 | (a,b,c) | | (a,b,c) | ST1 | (100) | LF-37 | L41-28/F |
| L33-28M | LMS-9 | (100) | | (a,b,c) | | | | | | |
| L34-45/F | LFS-10 | (100) | | (a,b,c) | | | | | | |
| L39-40/F | LFS-11 | (100) | | (a,b,c) | | | | | | |
| L42-50/M | LMS-12 | (100) | | (a,b,c) | | | | | | |
| L43-34/F | LFS-13 | (100) | | (a,b,c) | | | | | | |
| L44-38/F | LFS-14 | (100) | | (a,b,c) | | | | | | |
| L45-46/F | LFS-15 | (100) | | (a,b,c) | | | | | | |
| L35-24M | LMS-16 | (99.7) | | (a,b,c) | MJ99-116, monkey (AB107969) | (a,b,c) | | (99.7) | LM-38 | L19-20/M |
| L8-64/M | **LMS-17** | (99.7) | | **(a,b,c)** | | (a,b,c) | | (99.7) | LM-39 | L21-18/M |
| L2-17/M | **LMS-18** | (98.9) | ST2 | **(a,b,c)** | | (a,b,c) | ST2 | (98.9) | LM-40 | L6-27/M |
| L36-15/M | LMS-19 | (98.9) | | (a,b,c) | | (a,b,c) | | (98.9) | LF-41 | L14-27/F |
| L37-33/F | LFS-20 | (98.9) | | (a,b,c) | | (a,d) **(b,c)** | | (98.9) **(98.7)** | **LM-42** | L5-27/M |
| | | | | | | (a,b,c) | | (99.7) | LM-43 | L38-23/M |
| L7-25/M | **LMS-21** | (100) | | **(a,b,c)** | PJ99-162, pig (AB107963) | | | | | |
| L11-31/F | LFS-22 | (100) | | (a,b,c) | | | | | | |
| L15-34/M | LMS-23 | (100) | | (a,b,c) | | | | | | |
| L23-34/M | LMS-24 | (100) | | (a,b,c) | | | | | | |
| L9-48/M | LMS-25 | (100) | ST3 | (a,b,c) | | | | | | |
| L10-28/M | **LMS-26** | **(99.1)** (100) | | **(a,b)**(c,d) | | | | | | |
| L29-30/M | LMS-27 | (100) | | (a,b,c) | | | | | | |
| L30-19/F | LFS-28 | (100) | | (a,b,c) | | | | | | |
| | | | | clones sequences with two *Blastocystis* subtypes | | | | | | |
| L27-45/M | **LMS-29** | **(98.9)** | ST1 | **(a,b)** | MJ99-424, monkey (AB107967) | | | | | |
| | | **(99.7)** | ST3 | **(c,d)** | PJ99-162, pig (AB107963) | | | | | |
| | | | | | MJ99-424, monkey (AB107967) | (a,b) | ST1 | (99.7) | **LM-44** | L3-20/M |
| | | | | | MJ99-116, monkey (AB107969) | **(c,d)** | ST2 | (98.3) | | |
| | | | | | MJ99-424, monkey (AB107967) | **(a,b)** | ST1 | (99.7) | **LF-45** | L4-33/F |
| | | | | | MJ99-116, monkey (AB107969) | (c,d) | ST2 | (98.3) | | |

Designation: Subtype of *Blastocystis* (ST), Libyan (L), female (F), male (M). *Blastocystis* isolate from Libyan male (LM), male with symptom (LMS), female (LF), female with symptom (LFS). Isolate clones sequences (bold font) and the reference isolates (accession number) were used in the phylogenetic analysis. Specimens L18-9/F and L5-27/M were co-infected with *Giardia intestinalis* and *Cryptosporidium* species, respectively.
